# Metabolomic Profiling, Box–Behnken Design-Based Optimization of Ultrasonic Extraction, and Skin Anti-Aging Potential of the Green Husk of *Juglans regia* L. as a Sustainable Natural Waste

**DOI:** 10.3390/molecules30214191

**Published:** 2025-10-27

**Authors:** Sıla Özlem Şener, Sabita Shaha, Sahar Sadigh Barazandeh, Ömer Şen, Engin Koçak, Tuğba Subaş, Şerife Nur Kıraç, Emirhan Nemutlu

**Affiliations:** 1 Department of Pharmacognosy, Gulhane Faculty of Pharmacy, University of Health Sciences, 06210 Ankara, Türkiye; sadighshr@gmail.com (S.S.B.); sennur4241@gmail.com (Ş.N.K.); 2 School of Pharmacy and Medical Sciences, University of Bradford, Bradford BD7 1AZ, UK; s.shaha2@bradford.ac.uk; 3 Department of Basic Sciences and Health, Hemp Research Institute, Yozgat Bozok University, 66900 Yozgat, Türkiye; omer.sen@bozok.edu.tr; 4 Department of Analytical Chemistry, Gulhane Faculty of Pharmacy, University of Health Sciences, 06210 Ankara, Türkiye; engin.kocak@sbu.edu.tr; 5 Department of Pharmacognosy, Faculty of Pharmacy, Karadeniz Technical University, 61080 Trabzon, Türkiye; tugbasubas@ktu.edu.tr; 6 Department of Analytical Chemistry, Faculty of Pharmacy, Hacettepe University, 06800 Ankara, Türkiye; enemutlu@hacettepe.edu.tr

**Keywords:** walnut, green extraction, phenolic compounds, elastase inhibition, waste valorization

## Abstract

The green husk of *Juglans regia* L. is rich in bioactive phytochemicals and exhibits various biological activities. This study aimed to investigate the skin anti-aging potential of the green husk of *J. regia* by determining the optimal extraction conditions using a Box–Behnken Design (BBD), targeting elastase inhibition, and by correlating variations in phenolic compounds identified through metabolomic analyses with changes in the extraction conditions. Ultrasonic-assisted extraction was employed along with natural deep eutectic solvents (NADES). Three levels of three independent variables (NADES/H_2_O ratio, temperature, and extraction time) were incorporated into the BBD. Phenolic compounds were determined semi-quantitatively using liquid chromatography–quadrupole time-of-flight mass spectrometry (LC-q-TOF/MS), while elastase inhibition was evaluated by spectroscopic methods. Quadratic response surface models were proposed based on the BBD model adequacy test using multiple regression analysis. It was determined that the optimum conditions for maximizing phenolic content and elastase inhibition were 99.79% ethanol, 51.30 °C temperature, and 49.58 min, in which case the desirability score was 1. Metabolomic analysis identified 41 phenolic compounds across 27 ontological groups, with 24 compounds showing a semi-quantitative increase. Consequently, the waste green husk of *J. regia* demonstrated enhanced anti-aging potential due to the higher content and diversity of phenolic compounds.

## 1. Introduction

The World Health Organization (WHO) defines healthy aging as the ability to develop and maintain functional capacity healthily despite advancing age [1]. Skin aging, as a component of aging, results from changes that occur at the organ, tissue, and cellular levels. Unlike other age-related changes, skin aging is visibly apparent, which can have a more significant impact on human psychology, social relationships, and overall health [2,3]. The global market for anti-aging products has been expanding rapidly. In 2020, the market size in the United States of America (USA) was estimated at 34.2 billion dollars, with predictions indicating it will reach 47.8 billion dollars by 2027. China is expected to contribute 18.4 billion dollars to the global market by 2027. Other countries with notable shares in this market, although smaller in comparison, include Canada, Japan, Europe, and Germany [4].

Elastase is a protease that plays a significant role in skin aging, being a component of the chymotrypsin family. This enzyme is capable of degrading skin proteins such as elastin, collagen, and fibronectin [5]. Factors such as age and chronic exposure to ultraviolet (UV) radiation lead to increased elastase activity, which in turn exacerbates the degeneration of elastic fibers within the skin tissue. As this degeneration progresses, the skin loses its elasticity, resulting in the formation of wrinkles and sagging [6]. Consequently, there is a therapeutic interest in the use of elastase inhibitors for addressing skin aging [7].

In recent years, natural resources that have been classified as agricultural waste are gaining increasing significance due to their secondary metabolite content, which is associated with physiological activities. Their abundant availability and potential to contribute to environmental pollution when discarded without regulation have made them a focal point of numerous research studies. The demand for the utilization of naturally sourced agricultural waste in nutrition, food, cosmetics, pharmaceuticals, and industrial applications is rapidly increasing. One of the natural resources utilized in the generation of agricultural waste is the fruit of *Juglans regia* L. The outer green thick layer of *J. regia*, referred to as the green husk, is a significant agricultural by-product generated during the harvesting and processing of the fruit. This waste material has the potential to contribute to environmental pollution [8]. Traditionally, the green husks of *J.* regia have been reported to be utilized in the treatment of various ailments, including skin diseases, warts, stomach pain, and anemia [9,10]. The green husk of *J. regia* is particularly rich in phytochemical constituents, especially phenolic compounds. The primary chemical components of the green husk include naphthoquinones and their glycosides, diarylheptanoids, terpenes and their glycosides, flavonoids, sterols, and polyphenols. Furthermore, the green husk of *J. regia* has demonstrated antitumor, antioxidant, antiviral, and bacteriostatic activities [8,11,12,13]. It is important to use appropriate and environmentally friendly extraction methods to efficiently recover the bioactive compounds of *J. regia* green husks.

In contemporary research, the significance of green extraction techniques is increasingly recognized, as they aim to enhance extraction efficiency and quality by reducing extraction time, global energy consumption, solvent usage, environmental impact, economic costs, and waste production [14]. The fundamental principle of ultrasonic extraction, one of the green extraction methods, involves the mechanical, cavitational, and thermal effects that lead to the disruption of cell walls, resulting in smaller particle sizes and an increased mass transfer across cell membranes. The advantages of ultrasonic extraction include its safety and efficiency, rapid extraction kinetics, and the ability to extract heat-sensitive compounds [14,15,16,17]. The various organic solvents employed during extraction processes pose detrimental effects on both human health and the environment. This issue underscores the increasing significance of “green solvents” that can replace organic solvents derived from fossil fuels. Ionic liquids (ILs) and deep eutectic solvents (DES) are primary types of solvents classified as green solvents. ILs and DESs are frequently described as green solvent alternatives; however, their actual sustainability is still debated due to differences in toxicity, biodegradability, and environmental persistence. To enhance the safety of green solvents, natural deep eutectic solvents (NADES) have been developed by substituting the components in DESs with natural products that function as hydrogen bond donors (HBD). Numerous studies have demonstrated that NADES can effectively extract active compounds from a wide range of natural sources. The advantages of utilizing NADESs include ease of application, relatively low costs, ready-to-use formulations, and biodegradability [18].

Optimization studies aimed at determining the most suitable extraction conditions from plant sources with reduced time and energy expenditure have gained significant importance in contemporary applications. One of the frequently employed statistical optimization methods that incorporates both dependent and independent variables is the response surface methodology. This methodology utilizes a combination of statistical and mathematical techniques to derive the best statistical estimates from a limited set of experimental data [19]. Among the techniques utilized within response surface methodologies, the Box–Behnken design (BBD) is a second-order multivariate technique based on three-level factorial designs, which is employed to identify optimal experimental conditions. The number of experiments required for the development of the Box–Behnken technique (N) is defined by the equation N = 2k (k − 1) + C0, where k represents the number of factors and (C0) denotes the number of repetitions at the center point. By leveraging the Box–Behnken technique, it is possible to determine the optimal extraction conditions through statistical analysis using a limited set of experimental data [20,21].

Metabolites are low-molecular-weight organic molecules that participate in diverse biochemical reactions and represent intermediate or end-products of various cellular processes. Metabolomics has emerged as a pivotal discipline that investigates the complete set of metabolites within biological systems through an integrative and systems-based approach. One of the major applications of metabolomic studies is the discovery of novel bioactive molecules or drug candidates derived from natural sources. Beyond identifying such compounds, metabolomic analyses also provide insights into the interactions between specific metabolites-such as phenolic constituents-and biological systems. These analyses enable the comprehensive characterization of metabolites involved in these interactions and elucidate the underlying biochemical mechanisms [22]. Furthermore, advanced correlation analyses following metabolomic profiling allow the establishment of relationships between metabolic signatures and specific biological activities, offering a modern strategy to identify compounds responsible for the observed effects. The regression coefficients obtained from these correlation models indicate whether the associations between metabolites and activities are positive or negative—reflecting activating or inhibitory effects—and their magnitude provides an estimate of the strength of these interactions [23,24].

Phenolic compounds possess the potential to be utilized in the prevention and treatment of visible signs of both early and chronological aging, as well as age-related skin disorders, through various mechanisms in which they act as elastase inhibitors [25].

Skin aging is closely associated with oxidative stress, inflammation, and the degradation of structural proteins such as collagen and elastin, processes in which elastase plays a critical enzymatic role. In this context, the development of sustainable extraction strategies for obtaining phenolic-rich bioactives from natural resources represents a promising avenue for anti-aging applications. Therefore, this study aims to explore the potential of biological waste from *J. regia* green husks by preparing eco-friendly extracts rich in phenolic constituents and evaluating their relevance to skin-aging mechanisms. To this end, green husks of *J. regia* were utilized to formulate a green solvent, which was then applied in ultrasound-assisted extraction (UAE) to obtain environmentally sustainable extracts. The elastase inhibitory effects of these extracts were determined in vitro using spectroscopic techniques, with inhibitory potency expressed as half maximal inhibitory concentration (IC_50_). Optimization of the green extraction process was subsequently performed to identify the most effective extraction parameters for maximizing bioactivity while minimizing energy and time consumption. Furthermore, a metabolomic analysis was conducted to elucidate the influence of extraction conditions on phenolic composition and to establish the relationship between the identified phenolic metabolites and elastase inhibition. For the metabolomic analyses, three distinct extracts were evaluated. The first was the BBD-optimized extract (U1), obtained under the optimal extraction conditions determined by the BBD model. The second, the maceration extract (M1), was prepared using the conventional maceration technique to allow comparison between traditional and modern extraction approaches. The third extract, the optimized extract at maceration temperature (U2), was produced under the same optimized conditions as U1, except that the extraction temperature was adjusted to that of maceration, in order to specifically assess the effect of temperature on extraction efficiency. This integrative approach provides both a scientific and practical framework for the valorization of *J. regia* residues in sustainable anti-aging formulations.

## 2. Results and Discussion

### 2.1. Results of Elastase Inhibition

The analysis of IC_50_ values revealed that the coefficient of determination (R^2^) of the dose–response curves constructed from log concentration versus percentage inhibition data exceeded 0.99, confirming the reliability and consistency of the measurements. Based on the 17 experimental runs established by the BBD, elastase inhibition values varied considerably, with IC_50_ values ranging from 1.16 µg/mL to 45.82 µg/mL (Table 1). These results indicate that extraction parameters had a measurable influence on enzyme inhibition.

The relationships between the extraction variables (NADES ratio, temperature, and time) and elastase inhibition were subsequently evaluated using the BBD (Section 2.2), followed by optimization studies presented in Section 2.4.

### 2.2. Analysis of Box–Behnken Design

BBD is a response surface methodology employed to optimize experimental variables utilizing three-level factorial designs [20]. Three independent variable levels, NADES ratio, temperature, and time, were employed to facilitate the BBD. The BBD methodology serves to elucidate the relationship between experimental and predictive outcomes [26]. One metric employed to assess this significance is the percent relative error, which is determined using the formula [((predictive data–experimental data)/predictive data) × 100] [27]. The experimental design of the BBD, along with the experimental data, predictive data, and the percent relative error values for all responses, is detailed in Table 1.

The analysis employed a sequential model sum of squares, model summary, and lack of fit tests across various model types, including quadratic, cubic, two-factor interaction (2FI), and linear models, to identify the most suitable model for each response and derive the corresponding regression equations through analysis of variance (ANOVA). Additionally, the assessment of model reliability was conducted using the coefficient of variance (CV%), R^2^, and the adjusted coefficient of determination (R^2^a) [28]. The findings indicated that the quadratic model was the most appropriate, demonstrating a significant *p*-value (*p* < 0.0001), as supported by the analysis of the sum of squares and the lack of fit test. In addition, R^2^ was used to evaluate the reliability of the model. The R^2^ value of 0.9886, being close to 1, supports the significance of the quadratic model (Table S1).

Table 2 presents the coefficient estimates and *p*-values for the response of elastase inhibition. The inhibition of elastase was found to be significantly influenced by the linear coefficients (A, B, and C), two quadratic coefficients (A^2^ and B^2^), and two interaction coefficients (AC and BC) (*p*< 0.05) (Table 2). The most substantial impact on elastase inhibition, as indicated by the IC_50_ value, was attributed to temperature (B), which exhibited a regression coefficient of 12.87 and a highly significant *p*-value (*p* < 0.0001). An increase in the NADES/H_2_O ratio (A) corresponded to a reduction in the IC_50_ value for elastase inhibition with a regression coefficient of −10.14 and a *p*-value of less than 0.0001. Conversely, time (C) exhibited an inverse relationship, with a regression coefficient of −2.28 and a *p*-value of 0.0233. Additionally, the two quadratic terms A^2^ (*p* = 0.0300) and B^2^ (*p* < 0.0001) showed significant effects, both having a direct proportional influence with regression coefficients of 2.95 and 10.90, respectively. The interaction term (AC) also had a significant direct effect on the IC_50_ value of elastase inhibition (*p* = 0.0147, regression coefficient = 3.59), as well as another interaction term (BC) with a regression coefficient of −5.99 and *p*-value of 0.001. In contrast, the quadratic terms (C^2^) and interaction terms (AB) did not demonstrate significant effects, as indicated by *p*-values greater than 0.05.

Consequently, following the removal of the insignificant terms, the regression equation was revised, resulting in the subsequent reduced Equation (1) for the IC_50_ value of elastase inhibition.

Elastase inhibition (IC_50_ value) = 9.83 − 10.54A + 12.87B − 2.28C + 3.59AC − 5.99BC + 2.95A^2^ + 10.90B^2^ (1)

**Figure 1:**
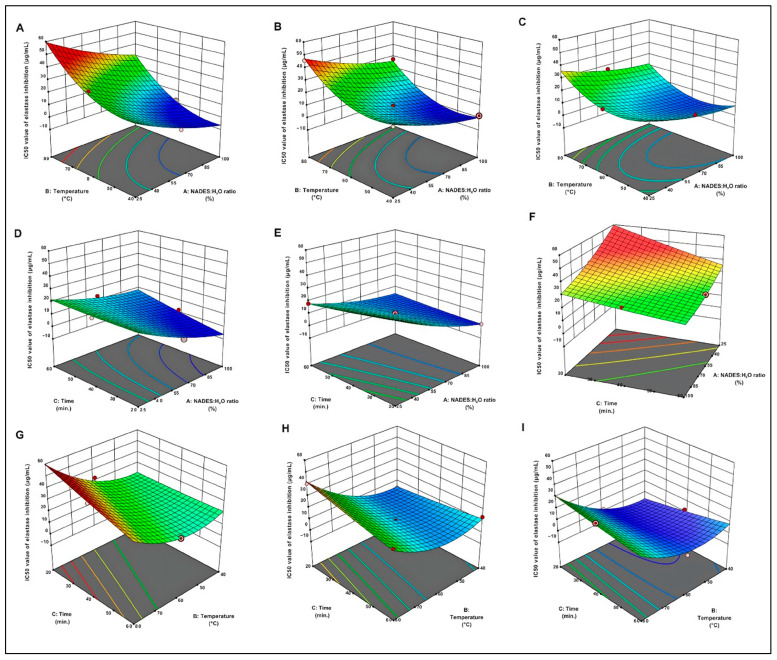
Three-dimensional Response Surface Graphs for IC_50_ Values of Elastase Inhibition Effect of Temperature: The relationship between the solvent ratio and different extraction times for the IC_50_ value of elastase inhibition: 20 min (**A**), 40 min (**B**), and 60 min (**C**). Effect of Time: The relationship between the solvent ratio and different temperatures for the IC_50_ value of elastase inhibition: 40 °C (**D**), 60 °C (**E**), and 80 °C (**F**). Effects of NADESs/H_2_O ratio: The relationship between the time and different temperatures for the IC_50_ value of elastase inhibition: 25% (**H**), 62.5% (**G**), and 100% (**I**).

The three-dimensional response surface plots provide a compelling visual representation of regression equations, illustrating the interplay between independent variables and the response variable [29]. As depicted in Figure 1, an increase in the NADES/H_2_O ratio resulted in a decrease in the IC_50_ value for elastase inhibition. Extraction durations between 40 and 60 min caused a significant increase in elastase inhibition. Furthermore, maintaining the extraction temperature within the range of 40 °C to 60 °C significantly lowered the IC_50_ value, thereby maximizing elastase inhibition.

#### 2.2.1. Effect of Extraction Temperature

Temperature is one of the most influential parameters governing the UAE, as it modulates cavitation behavior, solvent mobility, and the integrity of heat-sensitive bioactive compounds. A moderate increase in temperature enhances solvent diffusivity and softens plant tissue structures, thereby facilitating solute migration and improving extraction efficiency. In contrast, excessive heating can weaken cavitation collapse and accelerate the degradation of phenolic constituents, resulting in lower extraction yields and diminished bioactivity. Maintaining the extraction temperature within an intermediate range of approximately 40–60 °C has been shown to provide an effective compromise between enhanced mass transfer and preservation of molecular stability, ensuring sufficient energy for solvent permeation without compromising the structural integrity of active compounds [30,31].

Similar thermal patterns have been documented in various botanical matrices. For instance, *Benincasa hispida* seed oil extraction demonstrated an increase in yield with temperature up to about 52 °C, followed by a decline due to reduced cavitation efficiency. Under these optimized ultrasonic conditions, the extract exhibited considerably higher total phenolic content and antioxidant activity compared to the Soxhlet extract, highlighting that moderate UAE conditions improve both yield and functional compound recovery [32].

In agreement with these observations, the optimum extraction temperature obtained in this work (51.30 °C) falls well within the recommended thermal range, confirming that moderate heating promotes efficient cavitation and solvent diffusion without inducing thermal degradation. At this point, the extracts showed the highest total phenolic concentration and the strongest elastase inhibitory response, suggesting that phenolic compounds play a principal role in the anti-aging efficacy of the optimized extract. Similarly, *Peucedanum ostruthium* leaf extraction performed at 61 °C achieved maximum total phenolic (145.82 mg gallic acid equivalent-GAE/g dry weight-dw) and flavonoid (637.45 mg rutin equivalent-RE/g dw) levels, along with enhanced elastase inhibition [31].

Taken together, maintaining the extraction temperature within an intermediate range of temperature ensures an optimal equilibrium between ultrasonic mechanical disruption and phenolic compound stability, thereby maximizing both extraction yield and elastase-inhibitory potential.

#### 2.2.2. Effect of Extraction Time

The duration of UAE is a critical variable that governs both the rate of mass transfer of bioactive constituents and the intensity of cavitation occurring within the extraction medium. During the early stages of sonication, the formation and implosion of cavitation bubbles generate microjets that rupture plant cell walls, thereby facilitating solvent penetration and enhancing the transport of intracellular compounds. As extraction progresses, the diffusion of cellular metabolites into the solvent phase increases, resulting in a substantial improvement in extraction efficiency. However, when sonication exceeds the optimal point, localized heating and the accumulation of reactive radicals may occur, leading to oxidative degradation and conformational changes in thermolabile molecules. Hence, maintaining an intermediate extraction duration ensures a balanced environment that supports efficient mass transfer while preserving molecular stability [30,33].

Empirical evidence from diverse biological matrices indicates that UAE efficiency strongly depends on the optimization of both temperature and duration parameters. Moderate extraction times have been shown to enable complete cell disruption and effective solute diffusion without promoting chemical degradation. For instance, *Astragalus membranaceus* exhibited its highest flavonoid recovery at approximately 50 min and 58 °C, confirming the benefits of intermediate sonication periods [34]. Similarly, *Boletus bicolor* achieved maximal polyphenol extraction at 41 min and 40 °C [35]. In *Crocus sativus* by-products, extraction at 45 min and 45 °C produced the greatest phenolic content and antioxidant potential, whereas longer durations reduced both parameters [36]. The optimized extraction of polysaccharides from *Ficus microcarpa* aerial roots occurred at 49 min and 74 °C [37]. Likewise, fucoidan extraction from *Sargassum* species reached maximum antioxidant activity at 49 min and 50 °C, followed by a decline with prolonged exposure [38]. Additionally, the ultrasound-assisted recovery of flavonolignans from *Silybum marianum* (L.) Gaertn. Fruits were most efficient at 60 min and 45 °C, exhibiting strong collagenase and elastase inhibitory activity [39]. Within our optimized temperature range (40–60 °C), sonication durations of approximately 40–50 min appear to represent the most favorable conditions for maximizing the recovery of bioactive compounds from plant matrices while minimizing degradation.

Further studies have reported comparable tendencies. *Eisenia arborea* extracts displayed similar antioxidant activity at 60 and 90 min, yet elastase inhibition was markedly higher at 90 min, indicating that prolonged sonication can enhance the liberation of enzyme-inhibitory metabolites [40]. In contrast, *Phoenix dactylifera* L. leaves exhibited the highest phenolic and flavonoid yields, and the strongest, after a much shorter UAE duration of 29 min at 50 °C [41]. In another study, *Tamus communis* L. by-products extracted for 40 min at 70 °C exhibited significant increases in flavonoids and phenolic acids, leading to enhanced inhibition of skin-aging enzymes [42]. The influence of extraction time on elastase inhibition appears to vary depending on the plant matrix. This variability suggests that the effect is not solely time-dependent but also influenced by other extraction parameters, particularly temperature. Under extraction temperatures comparable to those applied in our experiments, the effective duration for maximizing elastase inhibition generally ranged between 40 and 60 min, which closely aligns with the extraction time determined in the present study. This observation is consistent with predictions from response surface methodology (RSM), confirming that moderate extraction durations provide an optimal balance between mass transfer efficiency and the preservation of thermolabile bioactives.

The optimized sonication time of 49.58 min identified in this study, in combination with metabolomic profiling, revealed an increased abundance of numerous bioactive metabolites, particularly phenolic derivatives. Metabolomic analyses further demonstrated that this extraction period enhanced the accumulation of phenolic metabolites and produced extracts characterized by greater chemical complexity and biological activity. Phenolic compounds are well established as potent secondary metabolites with significant elastase inhibitory capacity-a key mechanism underlying anti-aging effects. Therefore, the elevated levels of phenolic and other bioactive metabolites obtained under these optimized conditions likely contributed directly to the observed elastase inhibition. Collectively, these findings reinforce the general consensus that moderate ultrasonic durations facilitate efficient solvent–matrix interactions and cell disruption while preserving the structural and functional integrity of bioactive molecules.

#### 2.2.3. Effect of NADES/H_2_O Ratio

The deliberate incorporation of a controlled amount of water into the NADES system was intended to fine-tune its physicochemical behavior and improve extraction performance. Due to their dense hydrogen-bond network, NADESs typically exhibit high viscosity, which can impede solvent diffusion and mass transfer processes, thereby limiting the release of intracellular phytochemicals. The introduction of water partially weakens the hydrogen-bonding interactions between HBDs and hydrogen bond acceptors (HBAs), leading to a marked reduction in viscosity and an enhancement in solvent fluidity, which collectively facilitate deeper penetration of the solvent into the plant tissue [43,44]. In addition, a moderate water fraction promotes swelling of the lignocellulosic framework and expands the solvent’s polarity range, enabling improved solubilization of both hydrophilic and moderately lipophilic metabolites [45,46]. Conversely, when the water proportion exceeds a critical level, the supramolecular organization of NADES begins to collapse, compromising its characteristic solvating potential and disrupting the cooperative hydrogen-bond structure [47]. Therefore, maintaining an optimal water content is crucial for balancing viscosity reduction and solvent integrity, ensuring the highest extraction efficiency while preserving the distinctive hydrogen-bonding environment that defines NADES functionality. As previously reported, moderate water addition can decrease viscosity and enhance mass transfer, which may initially improve the extraction of especially phenolic compounds [43,44,46]. However, beyond a critical threshold of approximately 30–50% (*w*/*w*) H_2_O, the supramolecular hydrogen-bond structure of NADES begins to break down, reducing the solvent’s ability to dissolve phenolic acids and flavonoids [47,48,49,50,51]. Although water additions up to broad ranges (even approaching 95%) have been reported in some studies, such systems are generally considered to behave as aqueous solutions rather than true eutectic mixtures [46,47,48,49,50,51].

The solvent system containing a 100% NADES/H_2_O ratio achieved the highest elastase inhibitory response, indicating an optimal balance between solvation strength and structural stability that facilitated the efficient solubilization of phenolic compounds. In contrast, partially hydrated formulations (25% and 62.5% NADES/H_2_O) exhibited markedly lower inhibition, likely due to disruption of the hydrogen-bonding network within the choline chloride–glycerol matrix. The excessive water fraction weakened solvent–solute interactions and reduced extraction selectivity. These findings highlight the importance of maintaining a predominantly NADES-based composition while avoiding excessive water incorporation to preserve the cooperative hydrogen-bond structure and maximize elastase inhibition efficiency. Nevertheless, future studies employing a wider range of NADES/H_2_O ratios or alternative NADES compositions could provide a more comprehensive understanding of how water modulates solvent polarity, viscosity, and hydrogen-bond strength. Such investigations would allow the precise identification of the optimum water content that maximizes both extraction efficiency and biological activity across different plant matrices.

### 2.3. Validation of the Box–Behnken Design’s Accuracy

To ensure that the fitted model provides a reliable approximation of the actual values, it is essential to conduct a validation process [52]. The accuracy of the BBD was assessed through a comparison of diagnostic plots representing both the experimental and predicted model outcomes. As illustrated in Figure S1, these diagnostic plots demonstrated the model’s adequacy and the correlation between the experimental results and the predicted values concerning elastase inhibition. The proximity of the data points in the diagnostic plots to the reference straight line indicates a satisfactory fit between the experimental and model data. Consequently, the findings affirm that the Box–Behnken model is effective for evaluating elastase inhibition.

### 2.4. Optimisation of Extraction Procedures

**Figure 2:**
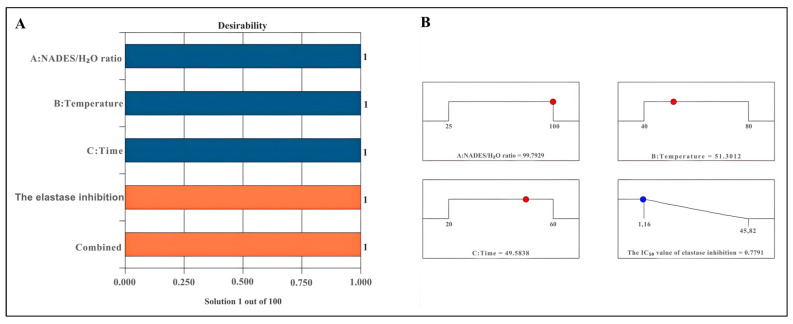
Desirability bar (**A**) and ramp for optimising extraction conditions (**B**). The blue bars represent the independent variables (A: NADES/H_2_O ratio, B: Temperature, and C: Time), while the orange bars indicate the dependent variables (Elastase inhibition and Combined response).

The desirability score serves as a critical metric for evaluating optimal conditions. A desirability score approaching 1 enhances the precision of the assessed conditions [53]. The proposed model delineates optimal conditions aimed at minimizing the desirability for elastase inhibition, thereby formalizing the model equation. The desirability scores recorded were 1 for elastase inhibition (Figure 2A). The optimal parameters identified for achieving the highest elastase inhibition effect included a NADES/H_2_O ratio of 99.79%, an extraction temperature of 51.30 °C, and an extraction duration of 49.58 min (Figure 2B).

It was determined that the experimental IC_50_ value of elastase inhibition (0.7735 ± 0.0182 μg/mL) and the predicted IC_50_ value of elastase inhibition (0.7791 ± 0.0275 μg/mL) for U1 were in close agreement. Additionally, the experimental and predicted values under other extraction conditions were found to correlate in terms of elastase inhibition. The IC_50_ value of U2 was observed to be 1.8213 ± 0.3173 μg/mL, whereas that of M1 was determined as 12.6377 ± 0.5275 µg/mL (Table 3).

### 2.5. Results of Metabolomics Analysis

Metabolomic analyses revealed the presence of 90 distinct compounds belonging to 58 different chemical classes in the samples. Among these compounds, it was identified that 41 are phenolic components classified into 27 different chemical categories (Table S2, Figure S2).

**Figure 3:**
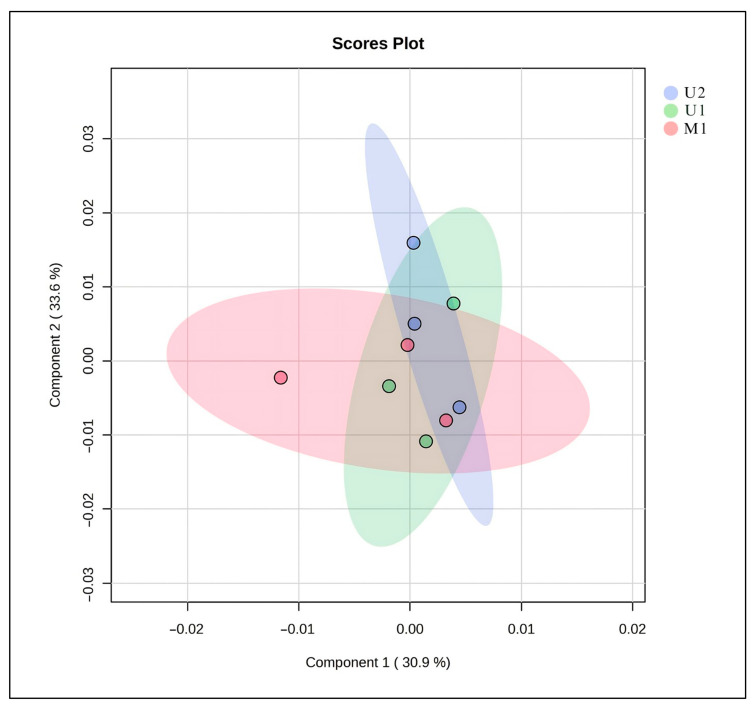
PCA of the metabolome structure of different extraction techniques. U1: The BBD-optimized extract, U2: The optimized extract at maceration temperature, M1: The maceration extract.

The Principal Component analysis (PCA) score plot illustrates the distribution of metabolite profiles obtained from three different extraction methods (U1, U2, and M1) (Figure 3). The first two principal components (PC1 and PC2) together explain 64.5% of the total variance in the dataset (30.9% and 33.6%, respectively). Samples from each extraction method form partially overlapping clusters, indicating that the methods yield comparable overall metabolite profiles. However, slight separation between groups suggests subtle differences in extraction efficiency and compound selectivity. The proximity of U1 and U2 clusters implies that these two methods isolate metabolites with similar polarity and chemical characteristics, whereas M1 shows a slightly broader distribution, reflecting a more diverse extraction pattern. Overall, the PCA results demonstrate that all three methods produce reproducible data, with U1 and U2 showing higher similarity in metabolite composition (Figure 3).

The objective of the correlation analyses was to investigate the impact of extraction conditions on the phytochemical profile. In this context, significant increases or decreases were observed in 88 out of 90 compounds analyzed across different extract groups (Table S2).

**Figure 4:**
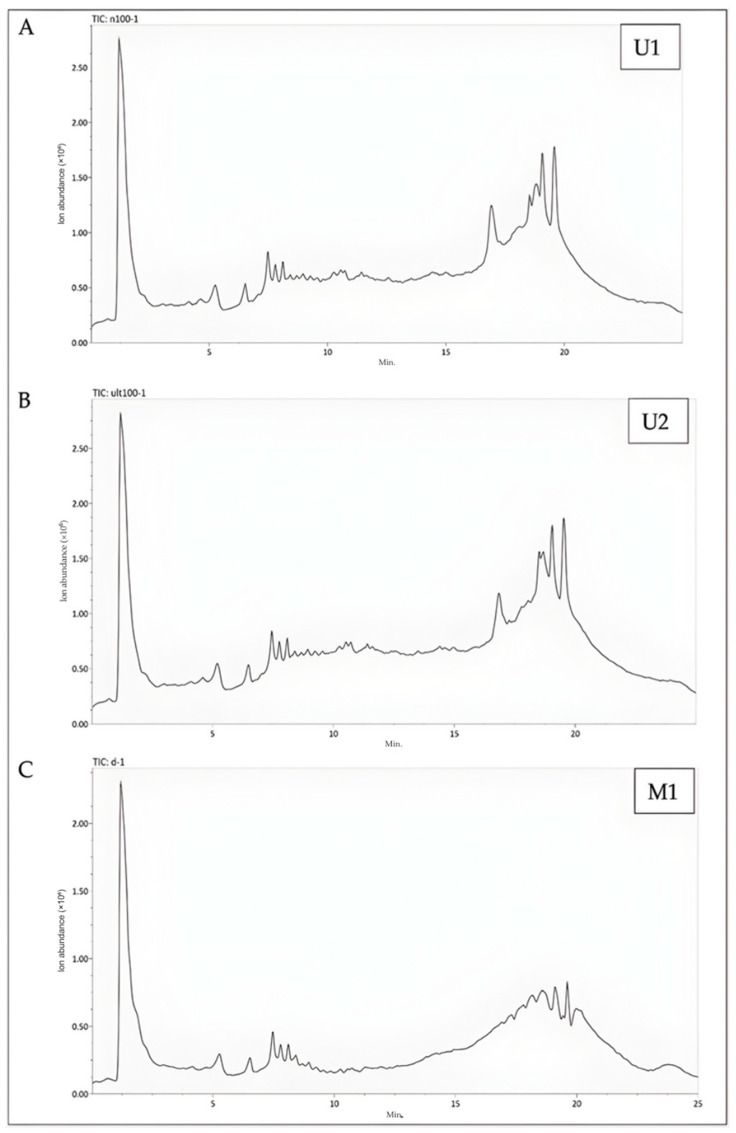
Representative LC-Q-TOF/MS chromatograms of samples. (**A**) The LC-Q-TOF/MS chromatogram of U1 (The BBD-optimized extract); (**B**) The LC-Q-TOF/MS chromatogram of U2 (The optimized extract at maceration temperature); (**C**) The LC-Q-TOF/MS chromatogram of M1 (The maceration extract).

The extraction conditions of U1 have been found to induce significant changes in 77 compounds when compared to the extraction conditions of M1. Specifically, the U1 extraction conditions resulted in a significant increase in 45 of these compounds, while a decrease was observed in 32 compounds. Among the 45 compounds, 24 were identified as having phenolic characteristics. In comparison to the total number of compounds, these extraction conditions led to a 26.67% increase in phenolic metabolites (Table S2, Figure 4A).

When U2 extraction conditions were compared with M1 extraction conditions, it was determined that they caused significant changes in 66 compounds. An increase was observed in 39 of these compounds, while a decrease was observed in 27. Of the 39 compounds with an increase, 27 were found to be phenolic in character. When compared to the total number of compounds under these extraction conditions, an increase of 26.67% was observed in the number of compounds with phenolic character (Table S2, Figure 4B).

The extraction conditions of U2 have been found to cause significant changes in 66 compounds when compared to the extraction conditions of M1. Among these compounds, an increase was observed in 39, while a decrease was noted in 27. It was determined that 27 of the 39 compounds exhibiting an increase possess phenolic characteristics. When compared to the total number of compounds under these extraction conditions, there was a 25.56% increase in the number of phenolic compounds (Table S2, Figure 4B).

When comparing the extraction conditions U1 and U2, it was observed that there were fewer changes in comparison to the maceration technique. A total of 23 compounds exhibited significant alterations. Among these compounds, 13 showed an increase under the U1 extraction conditions, with 6 of them identified as having phenolic characteristics. The U1 extraction conditions resulted in a 6.67% increase in the number of phenolic compounds relative to the total number of compounds (Table S2, Figure 4C).

It has been determined that neobonaspectin A, characterized by its sesquilignan structure, exhibits the highest increase in concentration when the extraction conditions are modified. The log(FC) values were established as 8 for U1/M1, −10 for M1/U2, and −2 for U2/U1 (Table S2). This indicates that the extraction conditions U1 and U2 significantly enhance the levels of neobonaspectin A.

It has been observed that the maceration technique is more effective for long-chain fatty acids. In the case of ultrasonic extraction, the similarity in temperature to the maceration method has resulted in an increased fatty acid ratio. However, a different scenario has been noted for the unsaturated fatty acid, linoleic acid. Under U2 extraction conditions, linoleic acid achieves the highest concentration, whereas the extract prepared using the maceration technique remains at a lower level (Table S2).

When comparing the extraction conditions of U1 with M1, it was observed that the compounds exhibiting the highest degree of variation after neobonaspectin A were, in order, phenolic herniarin, *β*-glucogallin, methyl gallate, and gallic acid. In the U2 extraction technique, this order was identified as herniarin, 2,5-dihydroxybenzoic acid, methyl gallate, and gallic acid. Furthermore, when the extraction conditions of U1 and U2 were compared, the compounds were identified as phelligridin I, D-quinic acid, and 2,5–dihydroxybenzoic acid (Table S2).

This study demonstrated that the UAE method produced substantially higher concentrations of several phenolic constituents compared to the conventional maceration process. Among the compounds that showed increased abundance, neubonaspectin A, herniarin, *β*-glucogallin, methyl gallate, and gallic acid were particularly prominent. The in silico molecular docking analysis revealed that herniarin, a coumarin derivative identified in *Mikania* sp., exhibits a notable interaction with the Ser195 residue located in the catalytic site of human neutrophil elastase (HNE). The calculated binding energy (−24.89 kcal/mol) of herniarin was comparable to that of other elastase-interacting phytochemicals such as psoralen and patuletin. The consistent engagement with the Ser195 residue suggests that herniarin may act as a moderate elastase modulator [54]. The activity-guided fractionation of the ethyl acetate extract derived from the pedicels and leaves of *Cotinus coggygria* Scop. yielded four bioactive constituents, among which methyl gallate demonstrated a moderate elastase inhibition (IC_50_ = 198.86 ± 0.84 µg/mL). This finding suggests that methyl gallate functions as a moderate yet biologically relevant elastase modulator, thereby contributing to the preservation of the extracellular matrix and underpinning the anti-aging potential of *C. coggygria* extracts [55]. The crude extract of white grape pomace, characterized by a high gallic acid content (596.36 ± 37.49 mg/kg dw), exhibited a marked ability to inhibit elastase activity in a dose-dependent manner, with an IC_50_ = 14.7 µg/mL [56]. To date, no scientific studies have been reported investigating the elastase inhibitory activity of either neubonaspectin A, *β*-glucogallin, or phelligridin I.

The compounds whose concentrations increased with the application of the UAE technique are known to exhibit anti-inflammatory, antioxidant, and epithelialization-promoting activities, thereby suggesting a potential contribution to anti-aging mechanisms. Certain compounds with a sesquilignan structure similar to neobonaspectin have been shown in nematode models to extend lifespan and enhance oxidative stress tolerance, potentially through the DAF-16-mediated signaling pathway [57]. Herniarin has been reported to suppress prostaglandin E2 (PGE2) release in a dose-dependent manner, reduce edema formation, and demonstrate that the substituent at the C-7 position plays a critical role in anti-inflammatory activity [58]. *β*-glucogallin, a potent aldose reductase inhibitor, has been shown to attenuate inflammatory responses and oxidative stress. *β*-glucogallin enhances skin barrier function by upregulating filaggrin and HAS3 expression in keratinocytes, and exerts anti-inflammatory effects by suppressing interleukin-4 (IL-4)/poly(I: C)-induced chemokines and pro-inflammatory cytokines [59,60]. Methyl gallate exerts its anti-aging potential primarily through reactive oxygen species (ROS) scavenging and activation of antioxidant pathways such as extracellular signal-regulated kinase 1/2 (ERK1/2)-nuclear factor erythroid 2-related factor 2 (Nrf2), and reduces the production of nitric oxide (NO) and IL-6 by macrophages [61]. Gallic acid inhibits pigmentation by downregulating melanogenic genes (tyrosinase, tyrosinase-related protein-1 (TRP-1), dopachrome tautomerase (Dct)) and suppressing microphthalmia-associated transcription factor (MITF) expression through inhibition of the cyclic adenosine monophosphate (cAMP)/protein kinase A (PKA)/cAMP-responsive binding protein (CREB) pathway, while activating ERK and AKT signaling, ultimately reducing melanin synthesis in cells, animal models, and zebrafish. These findings highlight its potential as an effective depigmenting agent for topical applications [62]. Phelligridin I, with its strong antioxidant properties, may play a role in modulating age-related cellular signaling changes [63]. Gentisic acid has been demonstrated to enhance keratinocyte proliferation by more than 20%, promote wound healing via ERK1/2 phosphorylation, and act as a safe tyrosinase inhibitor for the management of cutaneous hyperpigmentation disorders [64,65].

Although not as pronounced as the increases observed for the aforementioned compounds, several additional constituents that also exhibited elevated levels following extraction have been previously reported to possess elastase inhibitory activity in various scientific studies. Specifically, chlorogenic acid demonstrated significant elastase inhibition (IC_50_ = 203.3 µM). Molecular docking analysis revealed multiple hydrogen-bond interactions between chlorogenic acid and HNE residues Leu35, His40, Phe41, His57, and Gly193, resulting in a strong binding affinity. These molecular interactions support its potential role as a natural elastase regulator and highlight its possible contribution to anti-aging mechanisms [66]. Luteolin also exhibited potent inhibition of elastase release from human neutrophils, with an IC_50_ value of 6.91 ± 2.25 µM—comparable to that of the reference inhibitor LY294002 (IC_50_ = 4.97 ± 0.80 µM). This finding indicates that luteolin exerts a remarkable elastase-suppressing effect, likely contributing to its anti-inflammatory and dermoprotective properties through the attenuation of neutrophil-mediated proteolysis [67]. Ferulic acid showed measurable inhibitory effects on aging-associated enzymes, including elastase (IC_50_ = 75.61 µg/mL). This outcome suggests a moderate but functionally relevant elastase inhibition, implying that ferulic acid may help preserve skin elasticity by limiting elastin degradation, thereby enhancing its anti-aging potential [68]. Similarly, ellagic acid has been identified as a promising natural elastase inhibitor, exhibiting an IC_50_ value of 1.44 mg/mL. Spectroscopic analyses revealed that its interaction with elastase involves both static and dynamic quenching mechanisms, with complex formation at lower concentrations and dynamic processes predominating at higher levels. Thermodynamic evaluations further suggested that hydrogen bonding and van der Waals forces govern the interaction, leading to subtle conformational rearrangements in the enzyme structure [69]. Syringic acid also demonstrated a notable inhibitory effect on elastase activity (IC_50_ = 34.29 ± 1.71 µg/mL), indicating a moderate yet biologically relevant inhibition potential. This observation suggests that syringic acid may contribute to anti-aging and dermoprotective mechanisms by mitigating elastase-mediated extracellular matrix degradation [70].

The findings suggest that green extraction techniques not only provide environmental and economic advantages but also facilitate the recovery of bioactive phenolic compounds at concentrations biologically relevant for anti-aging applications. The optimization of extraction parameters enhances the correlation between phytochemical enrichment and biological efficacy, particularly in terms of enzyme inhibitory activities like elastase inhibition, which plays a critical role in preserving dermal elasticity and preventing extracellular matrix degradation. Thus, employing ultrasound-assisted green extraction under optimized conditions represents a sustainable and efficient strategy for maximizing the recovery of compounds with anti-inflammatory, antioxidant, epithelialization-promoting, and elastase-inhibitory properties. From a broader perspective, this integrative approach underscores how eco-friendly extraction technologies can bridge natural product chemistry and functional efficacy, paving the way for the development of scientifically grounded, sustainable anti-aging formulations that combine biological potency with environmental responsibility.

## 3. Materials and Methods

### 3.1. Raw Materials

The fruits of *J. regia* with green husk were harvested from Çamalanı, Akkuş, Ordu, Türkiye (40°49′41″ N 37°00′54″ E) (AEF26880). The green husks were carefully removed from the walnuts and subsequently dried to a stable weight in an incubator set at 35 °C for approximately 3 days. Following this process, the plant material was ground to approximately 40 mesh (0.42 mm), and the resulting homogeneous sample was stored in a desiccator, shielded from light, for future analyses [71].

### 3.2. Preparation of NADES

In the preliminary phase of this study, several NADES combinations were systematically prepared and evaluated at both 1:1 and 1:2 molar ratios between the HBA and HBD components. During this selection process, particular emphasis was placed on using naturally derived and biodegradable components that align with green chemistry principles. The formulations tested included ChCl:glucose (1:1, 1:2), ChCl:sucrose (1:1, 1:2), ChCl:lactic acid (1:1, 1:2), citric acid:sucrose (1:1, 1:2), and citric acid:glucose (1:1, 1:2) [72,73]. Among these, the ChCl:glycerol (1:2) system displayed the most favorable balance of physicochemical properties, superior phase homogeneity, and remarkable stability under UAE conditions. While certain sugar- and acid-based NADESs demonstrated inadequate solubilization potential, others with relatively acceptable solubility still suffered from elevated viscosity and poor thermal stability, factors that likely restricted effective cavitation and compromised overall extraction efficiency. In contrast, the ChCl:glycerol (1:2) mixture maintained a clear, low-viscosity phase and demonstrated structural stability during sonication, indicating its suitability as a thermally robust solvent. In particular, this system exhibited thermal stability within the operational temperature range applied during UAE, supporting its selection for subsequent optimization.

Consistent with our experimental findings, the ChCl:glycerol (1:2) NADES was selected due to its thermal stability, low viscosity, and biocompatibility compared with other tested systems. Its flexible hydrogen-bond network maintains structural integrity under ultrasonic and thermal conditions, ensuring efficient mass transfer and stable extraction performance [47,72]. The system also benefits from the GRAS status of components, providing a safe, low-cytotoxic, and highly biodegradable extraction medium suitable for applications in food, cosmetic, and pharmaceutical formulations [44,47,74]. Overall, these findings—supported by both preliminary solvent screening and literature evidence—confirmed the selection of the ChCl:glycerol (1:2) NADES as the optimal and most sustainable solvent system for the present study [43,75].

NADES was synthesized following the methodology described by Sakti et al. [18]. The preparation involved combining ChCl (CAS Number: 67-48-1, Cayman Chemical, ≥95%) as the HBA with glycerol (CAS Number: 56-81-5, Merck, ≥99.5%) as the HBD in a 1:2 (*w*/*w*) ratio. The mixture was stirred in a glass beaker using a magnetic stirrer (IKA^®^ C-MAG HS 7, IKA-Werke GmbH & Co. KG, Staufen, Germany) at 80 °C. The beaker was then sealed with Parafilm and left to rest for approximately one hour, or until a clear liquid was obtained, after which it was used immediately.

### 3.3. Preparations of Extracts with NADES

The extraction process utilizing NADES was conducted with an ultrasonicator (ISOLAB Ultrasonic C., Catalogue No:621.05.003, Eschau, Germany) operating at a power of 35 W and a frequency of 42.000 Hz. Powdered samples were placed in vials and combined with deionized water (dH_2_O) as outlined in Table 4. Subsequently, NADES was introduced into the vials. The UAE was carried out with different ratios of NADES-H_2_O mixture (25%, 62.5%, 100%), different temperatures (40 °C, 60 °C, 80 °C), and different times (20 min, 40 min, 60 min) (Table 4). Following this, the mixtures were transferred to centrifuge tubes and centrifuged for 10 min at a force of 3.283 g to facilitate the separation of the NADES liquid extract from the residual material. The resulting liquid layer was then filtered through a 0.45 μm Whatman micropore filter paper [18]. Subsequently, all extracts were subjected to evaporation under reduced pressure at 40 °C to yield crude extracts.

After the extraction process, the NADES phase was removed and treated as waste, since trace amounts of minor components could potentially remain and interfere with subsequent analyses. Nevertheless, due to its biodegradable nature and low environmental impact, the discarded solvent can still be regarded as an environmentally friendly system.

### 3.4. Evaluation of Elastase Inhibition

The elastase inhibitory activity was determined spectrophotometrically according to the method described by Moon et al. [76] and Sun et al. [66], with minor modifications. A volume of 50 µL of each extract solution, prepared at varying concentrations in 0.2 M Tris-HCl buffer (pH 7.8) containing 0.01% (*v*/*v*) dimethyl sulfoxide (DMSO), was mixed with 100 µL of HNE solution (17 mU/mL) prepared in the same buffer. For the control, 100 µL of HNE solution and 50 µL of buffer containing 0.01% DMSO were used without the addition of the extract. The blank contained 100 µL of enzyme-free phosphate buffer and 50 µL of phosphate buffer containing 0.01% DMSO. All tubes (sample, control, and blank) were incubated at 37 °C for 15 min. Subsequently, 50 µL of 5 mM N-succinyl-Ala-Ala-Ala-*p*-nitroanilide (STANA) substrate solution was added, and the mixtures were further incubated at 37 °C for 30 min. The absorbance values of the control and sample solutions were measured at 410 nm against the blank using a spectrophotometer. Catechin was used as the standard. The tests were conducted in triplicate, and necessary calculations were performed based on the average absorbance values. The elastase inhibition activity of the prepared plant extracts and the standard substance was calculated using the following Formula (2):

% inhibition = [(A_control_ − A_sample_)/A_control_] × 100 (2)

where A_control_ represents the absorbance value of the control solution at 410 nm, and A_sample_ denotes the absorbance value of the sample solution at the same wavelength. The IC_50_ value of the elastase enzyme was determined from the regression equation derived from the linear fit of the plotted data of concentration versus % elastase inhibition.

### 3.5. Optimization of Extraction Using Response Surface Methodology (RSM)

The optimization study was conducted utilizing a factorial-based BBD (3-factor, 3-level) as implemented in Design Expert V.8 [77]. This investigation focused on a dependent variable: elastase inhibition, while three independent variables: solvent ratio, temperature, and time were systematically analyzed. The selection of these factors and their corresponding levels was informed by prior research [78,79,80,81,82]. The coded values and their associated levels are presented in Table 4. The experimental design comprised a total of 17 runs, including five center points. The extraction conditions for all 17 runs are detailed in Table 1.

Also, to compare the extraction technique, the maceration process was similarly utilized with the same NADES/H_2_O ratios under optimal conditions at ambient temperature for one day, allowing for a comparison with both the green extraction method and the conventional technique.

### 3.6. Statistical Analysis

The tests were conducted in triplicate, and the results are expressed as mean ± standard deviation. IC_50_ values were determined from the regression equation of a linear curve plotted by the logarithm of the concentrations against the percentage inhibition values. Statistical analyses were performed using IBM SPSS Statistics software (Version 24.0) [83]. Principal Component Analysis (PCA) was performed to visualize the overall variance and identify clustering patterns among samples obtained from different extraction methods (U1, U2, and M1). PCA is an unsupervised multivariate statistical technique that reduces the dimensionality of complex datasets while retaining most of the variation present in the data [24]. In this study, PCA was applied to assess the reproducibility and discrimination capability of the extraction protocols. The first two principal components explained the majority of total variance, allowing the evaluation of similarities and differences in metabolite profiles across extraction methods.

### 3.7. Metabolomics Analysis

Metabolomics analyses were performed on the extracts derived from M1, U1, and U2. U1 was characterized as the extract obtained through ultrasonic extraction under optimal conditions, specifically aimed at elastase inhibition. While U2 refers to the extract prepared under optimal conditions with the same solvent ratio using the ultrasonic extraction technique, but at room temperature, M1 represents the extract prepared under optimal conditions with the same solvent ratio by the maceration technique for 24 h at room temperature.

Metabolite separation was performed using a Zorbax C18 column (1 × 50 mm, 1.8 μm, 100 Å) and analyzed through liquid chromatography–quadrupole time-of-flight mass spectrometry (LC-qTOF-MS) system (Agilent 6530). The mobile phase included solvent A (water containing 0.1% formic acid) and solvent B (acetonitrile with 0.1% formic acid), following a gradient elution method: 0–1 min, 10% B; 1–10 min, 10–90% B; 10–11 min, 90% B; 11–20 min, 90–10% B; 20–30 min, 10% B. The flow rate was set at 0.2 mL/min, with a 2 μL injection volume. The ESI source functioned in positive mode, with a capillary voltage of 4000 V and a capillary temperature of 300 °C. Auto MS-MS data acquisition covered an *m*/*z* range of 100 to 1700, with a minimum threshold of 200 counts. MS/MS fragmentation of plant metabolites was carried out at a collision energy of 20 eV.

Raw MS data were analyzed using MS-Dial version 2.56 for deconvolution, peak detection, and alignment [24]. The minimum peak height for detection was set at 2000 amplitude, with MS1 and MS2 tolerances of 0.01 and 0.025 Da, respectively. Molecular formula prediction and structural elucidation were performed using MS-FINDER version 3.04 [84]. Identifications were refined through MS/MS data analysis. Metabolites were matched against natural product databases, including the Universal Natural Products Database (UNPD), KNAPpSAcK, and PlantCyc, with a mass tolerance of 10 ppm. Only metabolites with a score higher than 6 were regarded as confidently identified.

## 4. Conclusions

This research aimed to determine the optimal extraction conditions for the green husk of walnuts by targeting elastase inhibition through BBD analysis. During the extraction process, environmentally friendly NADESs were employed. As a result of the study, the walnut green husk extract prepared through eco-friendly methods demonstrated a high elastase inhibition effect, revealing its anti-aging potential. Metabolomic analysis indicated an increase in phenolic compound content under the optimized conditions. The enhanced anti-aging potential was associated with an increase in both the number and rate of phenolic compounds. These findings emphasize the feasibility of implementing environmentally friendly and sustainable approaches through the valorization of natural waste, green extraction techniques, and process optimization. However, further research and clinical studies are required to support the efficacy of the green husk of walnuts in this regard.

## Figures and Tables

**Table 1:** Comparison of Experimental Data and Predictive Data Obtained by BBD.

	A	B	C	R1 (Elastase Inhibition)		
	%	°C	min.	Exp. (µg/mL)	Pre. (µg/mL)	PRE %
1	0	−1	−1	3.39	4.70	27.87
2	0	0	0	9.99	9.83	−1.63
3	0	0	0	9.96	9.83	−1.32
4	+1	−1	0	1.78	0.39	−346.34
5	−1	−1	0	19.61	21.24	7.67
6	−1	0	+1	18.06	17.99	−0.39
7	+1	0	−1	1.41	1.48	4.73
8	0	0	0	9.62	9.83	2.14
9	0	−1	+1	13.68	12.12	−12.87
10	0	0	0	9.72	9.83	1.12
11	0	+1	−1	40.85	42.41	3.68
12	−1	0	−1	32.67	29.73	−9.89
13	+1	0	+1	1.16	4.10	71.71
14	−1	+1	0	45.82	47.20	2.92
15	0	+1	+1	27.19	25.88	−5.06
16	+1	+1	0	27.52	25.89	−6.30
17	0	0	0	9.86	9.83	−0.31

Abbreviations: Exp.: experimental, Pre.: predictive, PRE: percent relative error, A: NADES/H_2_O ratio ((*w*/*w*); −1 = 25.00%, 0 = 62.50%, +1 = 100.00%); B: Temperature (°C; −1 = 40.00, 0 = 60.00, +1 = 80.00); C: Extraction time (min; −1 = 20.00, 0 = 40.00, +1 = 60.00).

**Table 2:** *p*-values and coefficient estimates of one response.

	Elastase Inhibition				
	*p* Value	Coefficient Estimate	Standard Error	95% CI Low	95% CI High
Model	<0.0001				
Intercept		9.83	0.9973	7.47	12.19
A-Solvent Ratio	<0.0001	−10.54	0.7884	−12.40	−8.67
B-Temperature	<0.0001	12.87	0.7884	11.00	14.73
C-Time	0.0233	−2.28	0.7884	−4.14	−0.4144
AB	0.9190	−0.1175	1.12	−2.75	2.52
AC	0.0147	3.59	1.12	0.9534	6.23
BC	0.0010	−5.99	1.12	−8.62	−3.35
A^2^	0.0300	2.95	1.09	0.3801	5.52
B^2^	<0.0001	10.90	1.09	8.33	13.47
C^2^	0.6314	0.5450	1.09	−2.02	3.11
Residual					
Lack of fit	<0.0001				

**Table 3:** Predicted and experimental values of optimal conditions and comparison with the conventional extraction technique (maceration).

U1		U2	M1
NADES/H_2_O ratio (*w*/*w*) → 99.79% Temperature (°C) → 51.30 Time (min.) → 49.58		NADES/H_2_O ratio (*w*/*w*) →99.79% Temperature (°C) → 25 Time (min.) → 49.58	NADES/H_2_O ratio (*w*/*w*) → 99.79% Temperature (°C) → 25 Time (min.) → 24 h
IC_50_ values of elastase inhibition			
Predicted	Experimental	Experimental	Experimental
0.7791 ± 0.0275 μg/mL	0.7735 ± 0.0182 μg/mL	1.8213 ± 0.3173 μg/mL	12.6377 ± 0.5275 μg/mL

U1: The BBD-optimized extract, U2: The optimized extract at maceration temperature, M1: The maceration extract.

**Table 4:** Independent and dependent variables of BBD Analysis.

Independent Variables					
Factors	Name	Units	Coded Low	Coded Mean	Coded High
A	NADES/H_2_O ratio *	%	−1 ↔ 25.00	0 ↔ 62.50	+1 ↔ 100.00
B	Temperature	°C	−1 ↔ 40.00	0 ↔ 60.00	+1 ↔ 80.00
C	Time	min.	−1 ↔ 20.00	0 ↔ 40.00	+1 ↔ 60.00
Dependent Variables					
Responses	Name	Units			
R1	Elastaz inhibition (IC_50_)	μg/mL			

* NADES/H_2_O (*w*/*w*).

## Data Availability

Data are contained within the article.

## Supplementary Materials

The following supporting information can be downloaded at: https://www.mdpi.com/article/10.3390/molecules30214191/s1, Table S1. Results of the BBD’s model adequacy test. Table S2. List of possible identified metabolites of UMS using ESI–MS/MS in the negative ionization mode ([M−H]^−^) and advanced correlation analysis data. Figure S1. Diagnostic plots for the Box–Behnken model accuracy of predicted and actual data against elastase inhibition. Predicted versus actual values for elastase inhibition in the Box–Behnken model. The color scale indicates the magnitude of the response, where blue represents lower inhibition levels and red represents higher inhibition levels. Figure S2. Hierarchical clustering heat map showing the differential distribution of metabolites among the extracts. The color scale represents normalized relative abundance values ranging from 1 (lowest abundance, blue) to +1 (highest abundance, red).

## Abbreviations

The following abbreviations are used in this manuscript:

WHO	World Health Organization
USA	United States of America
UV	Ultraviolet
ILs	Ionic liquids
DES	Deep eutectic solvents
NADES	Natural Deep Eutectic Solvents
HBD	Hydrogen bond donor
BBD	Box–Behnken design
UAE	Ultrasound-assisted extraction
IC_50_	Half maximal inhibitory concentration
U1	The BBD-optimized extract
M1	The Maceration extract
U2	The Optimized extract at maceration temperature
2FI	Two-factor interaction
R^2^	Coefficient of determination
PRE	Percent relative error
ANOVA	Analysis of variance
CV%	Coefficient of variance
R^2^a	Adjusted coefficient of determination
GAE	Gallic acid equivalent
dw	Dry weight
RE	Rutin equivalent
ChCl	Choline chloride
RSM	Response surface methodology
PCA	Principal Component Analysis
ROS	Reactive oxygen species
ERK1/2	Extracellular signal-regulated kinase 1/2
Nrf2	Nuclear factor erythroid 2–related factor 2
NO	Nitric oxide
HNE	Human neutrophil elastase
PGE2	Prostaglandin E2
IL	Interleukin
Dct	Dopachrome tatamerase
TRP-1	Tyrosinase-related protein-1
MITF	Microphthalmia-associated transcription factor
cAMP	Cyclic adenosine monophosphate
CREB	cAMP-responsive binding protein
dH_2_O	Deionized water
HBA	Hydrogen bond acceptor
DMSO	Dimethyl sulfoxide
STANA	N-succinyl-ala-ala-ala-*p*-nitroanilide
LC-qTOF-MS	Liquid chromatography–quadrupole time-of-flight mass spectrometry
UNPD	Universal Natural Products Database
