# Supplementary material for: Metabolomic Profiling, Box–Behnken Design-Based Optimization of Ultrasonic Extraction, and Skin Anti-Aging Potential of the Green Husk of Juglans regia L. as a Sustainable Natural Waste
Source: Molecules. 2025 Oct 27;30(21):4191. doi: 10.3390/molecules30214191 (PMC12608389; doi:10.3390/molecules30214191)
Supplement: Supplementary file 1 [file molecules-30-04191-s001.zip › molecules-3895599-supplementary.pdf]

molecules

MDPI

Supplementary Material

Metabolomic Profiling, Box–Behnken Design-Based Optimization of Ultrasonic Extraction, and Skin Anti-Aging Potential of the Green Husk of *Juglans regia* L. as a Sustainable Natural Waste

Sıla Özlem Şener [1,*], Sabita Shaha [2], Sahar Sadigh Barazandeh [1], Ömer Şen [3], Engin Koçak [4], Tuğba Subaş [5], Şerife Nur Kıraç [1] and Emirhan Nemutlu [6]

[1] Department of Pharmacognosy, Gulhane Faculty of Pharmacy, University of Health Sciences, Ankara, Türkiye; silaozlem.sener@sbu.edu.tr (S.Ö.Ş.); Sadighshr@gmail.com (S.S.B.); sennur4241@gmail.com (Ş.N.K.)
[2] School of Pharmacy and Medical Sciences, University of Bradford, Bradford, UK; s.shaha2@bradford.ac.uk
[3] Department of Basic Sciences and Health, Hemp Research Institute, Yozgat Bozok University, Yozgat, Türkiye; omer.sen@bozok.edu.tr
[4] Department of Analytical Chemistry, Gulhane Faculty of Pharmacy, University of Health Sciences, Ankara, Türkiye; engin.kocak@sbu.edu.tr
[5] Department of Pharmacognosy, Faculty of Pharmacy, Karadeniz Technical University, Trabzon, Türkiye; tugbasubas@ktu.edu.tr
[6] Department of Analytical Chemistry, Faculty of Pharmacy, Hacettepe University, Ankara, Türkiye; enemutlu@hacettepe.edu.tr
* Correspondence: silaozlem.sener@sbu.edu.tr

Table S1. Results of the BBD's model adequacy test.

Source	Sum of Squares	df	Mean Square	*F*-value	*p*-value	Remark
Sequential model: Sum of squares of elastase inhibition						
Mean vs Total	4687.51	1	4687.51			
Linear vs Mean	2253.71	3	751,24	12.36	0.0004	
2FI vs Linear	195.01	3	65,00	1.09	0.3967	
**Quadratic vs 2FI**	**560.23**	**3**	**186.74**	**37.55**	**0.0001**	**Suggested**
Cubic vs Quadratic	34.71	3	11.57	464.69	< 0.0001	Aliased
Residual	0.0996	4	0.0249			
Total	7731.27	17	454.78			
Lack of fit tests of elastase inhibition						
Linear	789.95	9	87.77	3524.99	< 0.0001	
2FI	594.94	6	99.16	3982.21	< 0.0001	
**Quadratic**	**34.71**	**3**	**11.57**	**464.69**	**< 0.0001**	**Suggested**
Cubic	0.0000	0				Aliased
Pure Error	0.0996	4	0.0249			

Table S1. *Cont.*

Source	Std. Dev.	R^2	Adjusted R^2	Predicted R^2	PRESS	Remark
Linear	7.80	0.7404	0.6805	0.5294	1432.34	
2FI	7.71	0.8045	0.6872	0.2980	2136.67	
**Quadratic**	**2.23**	**0.9886**	**0.9739**	**0.8175**	**555.56**	**Suggested**
Cubic	0.1578	1.0000	0.9999		*	Aliased

Table S2. List of possible identified metabolites of UMS using ESI–MS/MS in the negative ionization mode ($[M–H]^-$) and advanced correlation analysis data

			U2/U1		M1/U2		U1/M1	
**Metabolite name**	**Ontology**	**Average Mz**	**Fold Change**	**log_2(FC)**	**Fold Change**	**log_2(FC)**	**Fold Change**	**log_2(FC)**
**1,2-benzenediol**	Catechols	109.0284	*	*	*	*	1.2741	0.3495
**14beta-15alpha-dihydroxy-delta-4pregnene-3, 20 dione**	Gluco/mineralocorticoid, progestogins and derivatives	345.2039	1.5855	0.6650	*	*	1.4913	0.5766
**18-Hydroxyoctadecanoic acid**	Long-chain fatty acids	299.2605	0.6305	-0.6655	5.7343	2.5196	0.1099	-3.1851
**1-Hydroxyanthraquinone**	Anthraquinones	223.0418	*	*	*	*	0.7477	-0.4196
**1-*O*-caffeoylglucose**	Hydroxycinnamic acid glycosides	341.0850	*	*	0.7799	-0.3586	1.2116	0.2769
**2,5-dihydroxybenzoic acid**	Hydroxybenzoic acid derivatives	153.0212	0.7875	-0.3447	0.5421	-0.8834	1.4526	0.5387
**2beta, 3beta, 4beta-trihydroxypregnan-16-one**	Gluco/mineralocorticoids, progestogins and derivatives	349.2354	*	*	2.0780	1.0552	0.4085	-1.2918
**3-(4-hydroxy-3-methoxyphenyl)-prop-2-enoic acid**	Hydroxycinnamic acids	193.0505	*	*	0.8329	-0.2638	1.2490	0.3208
**6-(4-carboxyphenoxy)-3, 4, 5-trihydroxyoxane-2-carboxylic acid**	Phenolic glycosides	313.0560	*	*	1.4489	0.5350	0.7594	-0.3970
**6, 6'-di-tert-butyl-2,2'-methylenedi-*p*-cresol**	Diphenylmethanes	339.2317	*	*	1.9819	0.9869	0.4647	-1.1056
**6''-malonylcosmosiin**	Flavonoid-7-*O*-glycosides	517.0938	*	*	1.2510	0.3231	0.7409	-0.4327
**7-hydroxy-4-methylcoumarin**	7-hydroxycoumarins	175.0397	*	*	0.7751	-0.3675	1.4532	0.5393
**Alpha-dimorphecolic acid**	Lineolic acids and derivatives	295.2280	*	*	1.2929	0.3706	0.7632	-0.3899

Table S2. *Cont.*

**Amicoumacin A**	Beta amino acids and derivatives	422.1942	*	*	0.5965	-0.7453	1.8263	0.8689
**Anhydrofusarubin lactone**	Isochromanequinones	301.0329	*	*	1.2191	0.28582	0.7361	-0.4420
**Anthoptilide D;(+)-Anthoptilide D**	Diterpene lactones	491.2080	*	*	0.7818	-0.3552	1.2637	0.3376
**Arachidic acid**	Long-chain fatty acids	311.2937	0.6721	-0.5733	1.2223	0.2896	0.5498	-0.8629
**Artemetin**	7-*O*-methylated flavonoids	387.1081	*	*	0.7763	-0.3652	1.3105	0.3901
**Astilbin**	Flavonoid-3-*O*-glycosides	449.1091	*	*	0.8204	-0.2856	*	*
**Aucubin**	Iridoid *O*-glycosides	345.1172	1.2274	0.2956	*	*	*	*
**Auraptene**	Terpene lactones	297.1520	*	*	*	*	0.8314	-0.2664
**Ayapin**	Coumarins and derivatives	189.0201	*	*	0.7915	-0.3373	1.2902	0.3676
**Beta-glucogallin**	Tannins	331.0644	*	*	0.6398	-0.6455	1.7186	0.7812
**Bis(glycerophospho)glycerol**	Glycerophosphoglycerophosphoglycerols	399.0475	1.2044	0.2683	*	*	1.3262	0.4074
**Bisosthenon B**	Cyclobutane lignans	487.1386	*	*	0.8321	-0.2651	*	*
**Catechin 7-glucoside**	Flavonoid-7-*O*-glycosides	451.1281	*	*	*	*	1.2286	0.2970
**Centaurein**	Flavonoid-7-*O*-glycosides	521.1309	*	*	0.8182	-0.2894	*	*
**Chlorogenic acid**	Quinic acids and derivatives	353.0859	*	*	0.7380	-0.4383	1.2982	0.3765
**D-(-)-Quinic acid**	Quinic acids and derivatives	191.0508	0.6676	-0.5829	*	*	0.5938	-0.7518
**Decarbamoylgonyautoxin III**	Saxitoxins, gonyautoxins, and derivatives	351.0709	*	*	0.8000	-0.3219	1.2021	0.2656
**Drosophylloside;(-)-Drosophylloside**	Phenolic glycosides	379.1040	1.2039	0.2678	*	*	1.3187	0.3991
**Ellagic acid**	Hydrolyzable tannins	300.9993	*	*	*	*	1.2347	0.3042
**Emodic acid**	Anthracenecarboxylic acids	299.0205	*	*	*	*	0.7943	-0.3322
**Fonsecin**	Naphthopyranones	289.0707	*	*	0.7462	-0.4224	1.2982	0.3765
**Fulvic acid**	Chromones	307.0455	*	*	1.2474	0.3190	0.8082	-0.3073
**Fumaric acid**	Dicarboxylic acids and derivatives	115.0039	*	*	0.5678	-0.8165	1.6978	0.7637

Table S2. *Cont.*

**Furospongin**	Aromatic monoterpenoids	325.1827	1.4217	0.5076	1.3270	0.4082	*	*
**Gallic acid**	Gallic acids	169.0149	*	*	0.5641	-0.8260	1.6375	0.7115
**Globularin**	*O*-glycosyl compounds	491.1511	*	*	0.7915	-0.3374	*	*
**Glyceric acid**	Sugar acids and derivatives	105.0204	*	*	0.5630	-0.8287	1.9395	0.9556
**Gossypetin 7-methyl ether 8-acetate**	Flavonols	373.0573	*	*	1.3548	0.4381	0.6417	-0.6401
**Guibourtinidol-(4alpha->2)-3,5,3',4'-tetrahydroxystilbene**	Linear diarylheptanoids	499.1399	*	*	0.6332	-0.6594	1.5884	0.6676
**Heptadecanoic acid**	Long-chain fatty acids	269.2465	1.4537	0.5397	2.4585	1.2978	0.5913	-0.7581
**Herniarin**	Coumarins and derivatives	175.0399	*	*	0.5324	-0.9095	2.1109	1.0779
**Hispidulin 7-*O*-beta-glucoside**	Flavonoid-7-*O*-glycosides	461.1055	*	*	*	*	*	*
**Homovanillic acid**	Methoxyphenols	181.0496	*	*	0.7432	-0.4281	1.3951	0.4804
**Hyrtiosenolide A**	Ketals	277.1418	*	*	*	*	*	*
**Hyrtiosenolide B**	Ketals	277.1437	0.8082	-0.3073	*	*	0.8215	-0.2836
**Isofraxidin**	7-hydroxycoumarins	221.0458	*	*	1.3430	0.4254	0.7915	-0.3373
**Isoplumericin**	Terpene lactones	289.0719	*	*	0.7373	-0.4396	1.3970	0.4824
**Isoquercitrin**	Flavonoid-3-*O*-glycosides	463.0873	*	*	0.8034	-0.3158	1.2176	0.28401
**Itaconic acid**	Branched fatty acids	129.0204	*	*	0.7018	-0.5108	1.4128	0.4985
**Juferin**	Sesquiterpenoids	339.1977	*	*	1.2266	0.2947	*	*
**Kaempferol 3-(6''-acetylglucoside)-7-glucoside**	Flavonoid-7-*O*-glycosides	651.1580	0.8218	-0.2831	*	*	*	*
**L-beta-homotyrosine**	Beta amino acids and derivatives	194.0876	*	*	0.5746	-0.7995	1.9197	0.9409
**Linoleic acid**	Lineolic acids and derivatives	280.4472	1.7269	0.7882	*	*	2.0546	1.0389
**L-lanthionine**	L-cysteine-S-conjugates	207.0437	*	*	6.0586	2.5990	0.1669	-2.5828
**L-Malic acid**	Beta hydroxy acids and derivatives	133.0147	*	*	0.5760	-0.7957	1.7918	0.8414
**Luteolin**	Flavones	285.0413	*	*	1.2447	0.3158	*	*
**Maleic acid**	Dicarboxylic acids and derivatives	115.0039	*	*	0.5579	-0.8419	1.8455	0.8840
**Mayoside**	Anthracenes	523.1216	*	*	*	*	0.8187	-0.2886

Table S2. *Cont.*

**Medicagol**	Coumestans	295.0250	*	*	*	*	1.2200	0.2868
**Methyl (9Z)-10'-oxo-6, 10'-diapo-6-carotenoate**	Fatty aldehydes	311.1668	*	*	*	*	0.8276	-0.2729
**Methyl gallate**	Galloyl esters	183.0306	*	*	0.5484	-0.8668	1.7182	0.7809
**Methyl 4-*O*-galloylchlorogenate**	Quinic acids and derivatives	519.1127	*	*	0.6875	-0.5405	1.3988	0.4842
**MG(16:0/0:0/0:0)**	1-monoacylglycerols	329.2676	0.3559	-1.4906	2.2503	1.1701	0.1582	-2.6607
**Muscapurpurin**	Tetracarboxylic acids and derivatives	417.0577	*	*	0.1967	-2.3457	5.3659	2.4238
**Myricetin 3-arabinoside**	Flavonoid-3-*O*-glycosides	449.0692	*	*	*	*	0.79038	-0.3394
**Myristic acid**	Long-chain fatty acids	227.2020	*	*	3.4640	1.7924	0.2750	-1.8624
**Neobonaspectin A;(+)-Neobonaspectin A**	Lignans, neolignans and related compounds	653.2937	0.2500	-2	0.0006	-10.7000	415.9800	8.7004
**Okanin 4-methyl ether 4'-(6''-acetylglucoside)**	Flavonoid *O*-glycosides	505.1349	0.7914	-0.3375	*	*	*	*
**Oleic acid**	Long-chain fatty acids	281.2472	0.80538	-0.3122	3.9903	1.9965	0.2018	-2.3088
**Palmitic acid**	Long-chain fatty acids	255.2333	1.2138	0.2795	3.8044	1.9277	0.3191	-1.6481
**Palmitoleic acid**	Long-chain fatty acids	253.2167	0.6561	-0.6080	3.0671	1.6169	0.2139	-2.2249
**Pantothenic acid**	Secondary alcohols	218.1041	*	*	0.7850	-0.3492	1.3775	0.4620
**Pentadecanoic acid**	Long-chain fatty acids	241.2175	1.4735	0.5593	3.5475	1.8268	0.4154	-1.2676
**Petroselinic acid**	Long-chain fatty acids	281.2483	*	*	2.2738	1.1851	0.4475	-1.1602
**Phelligridin I**	Isocoumarins and derivatives	623.0839	0.6540	-0.6126	0.7313	-0.4515	*	*
**Phlorizin**	Flavonoid *O*-glycosides	435.1299	*	*	0.6742	-0.5688	1.3355	0.4173
***p*-Hydroxymethoxybenzobijuglone**	Pentacenequinones	415.0439	*	*	1.2527	0.3251	0.7991	-0.3235
**Protocatechuic acid**	Hydroxybenzoic acid derivatives	153.0204	*	*	0.7081	-0.4979	1.4210	0.5069
**Protodestruxin**	Cyclic depsipeptides	564.3386	0.2976	-1.7487	3.9512	1.9823	0.0753	-3.731
**Rubellin**	Bufanolides and derivatives	619.2392	*	*	*	*	1.2156	0.2816

Table S2. *Cont.*

**Sclerophytin F methyl ether**	Eunicellane and asbestinane diterpenoids	351.2510	2.5735	1.3637	0.4480	-1.1585	5.7447	2.5222
**Stearic acid**	Long-chain fatty acids	283.2645	2.6140	1.3862	1.2217	0.2889	2.1397	1.0974
**Stryspinolactone**	Naphthopyranones	271.0822	*	*	0.7514	-0.4124	1.3237	0.4046
**Succinic acid**	Dicarboxylic acids and derivatives	117.0201	*	*	0.4980	-1.0059	2.1870	1.1290
**Syringic acid**	Gallic acid and derivatives	197.0461	*	*	1.3211	0.4018	0.6770	-0.5629
**Thelephoric acid**	Benzofurans	351.0139	*	*	*	*	0.8094	-0.3050
***trans*-Ferulic acid**	Hydroxycinnamic acids	193.0511	*	*	*	*	1.2075	0.2719

*Significant changes not detected. U1: The BBD-optimized extract, M1: The maceration extract, U2: The optimized extract at maceration temperature.

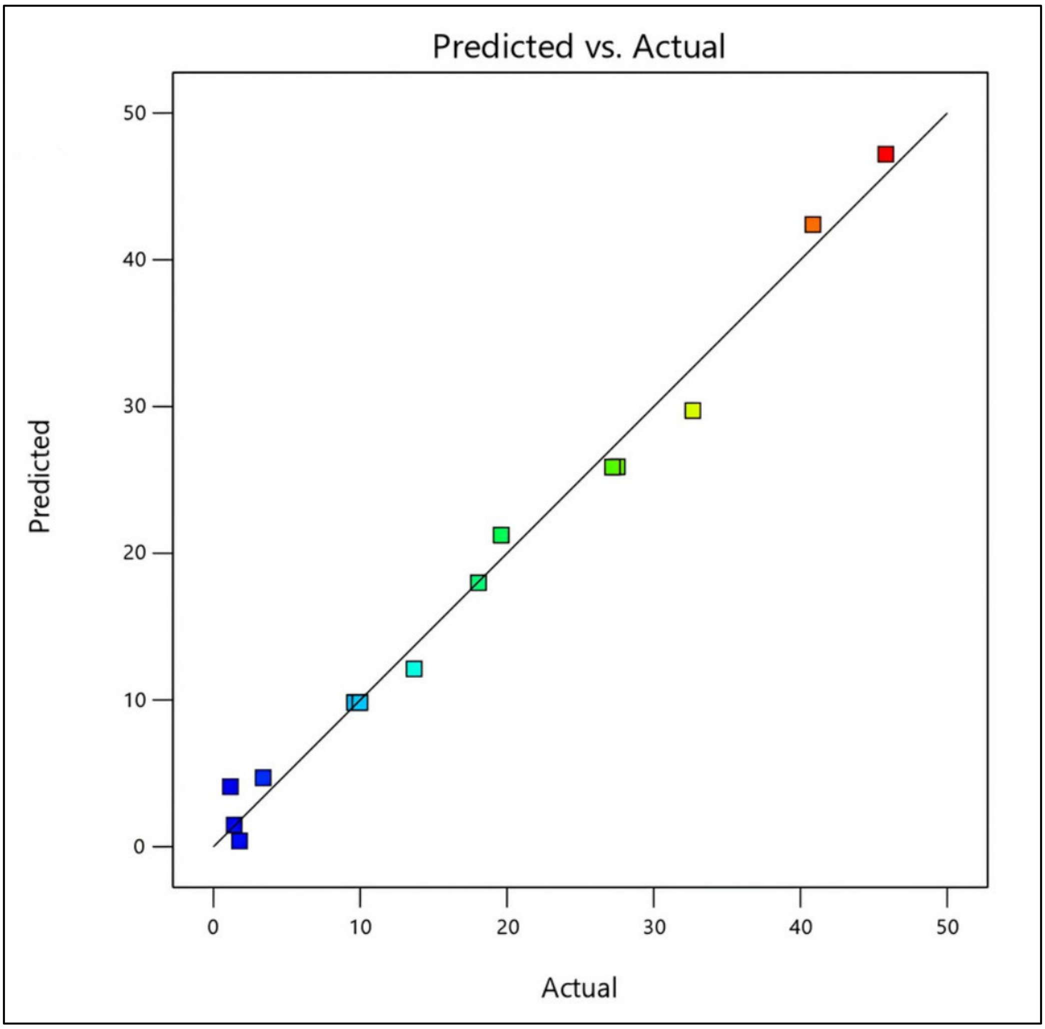

Figure S1. Diagnostic plots for the Box–Behnken model accuracy of predicted and actual data against elastase inhibition. Predicted versus actual values for elastase inhibition in the Box–Behnken model. The color scale indicates the magnitude of the response, where blue represents lower inhibition levels and red represents higher inhibition levels.

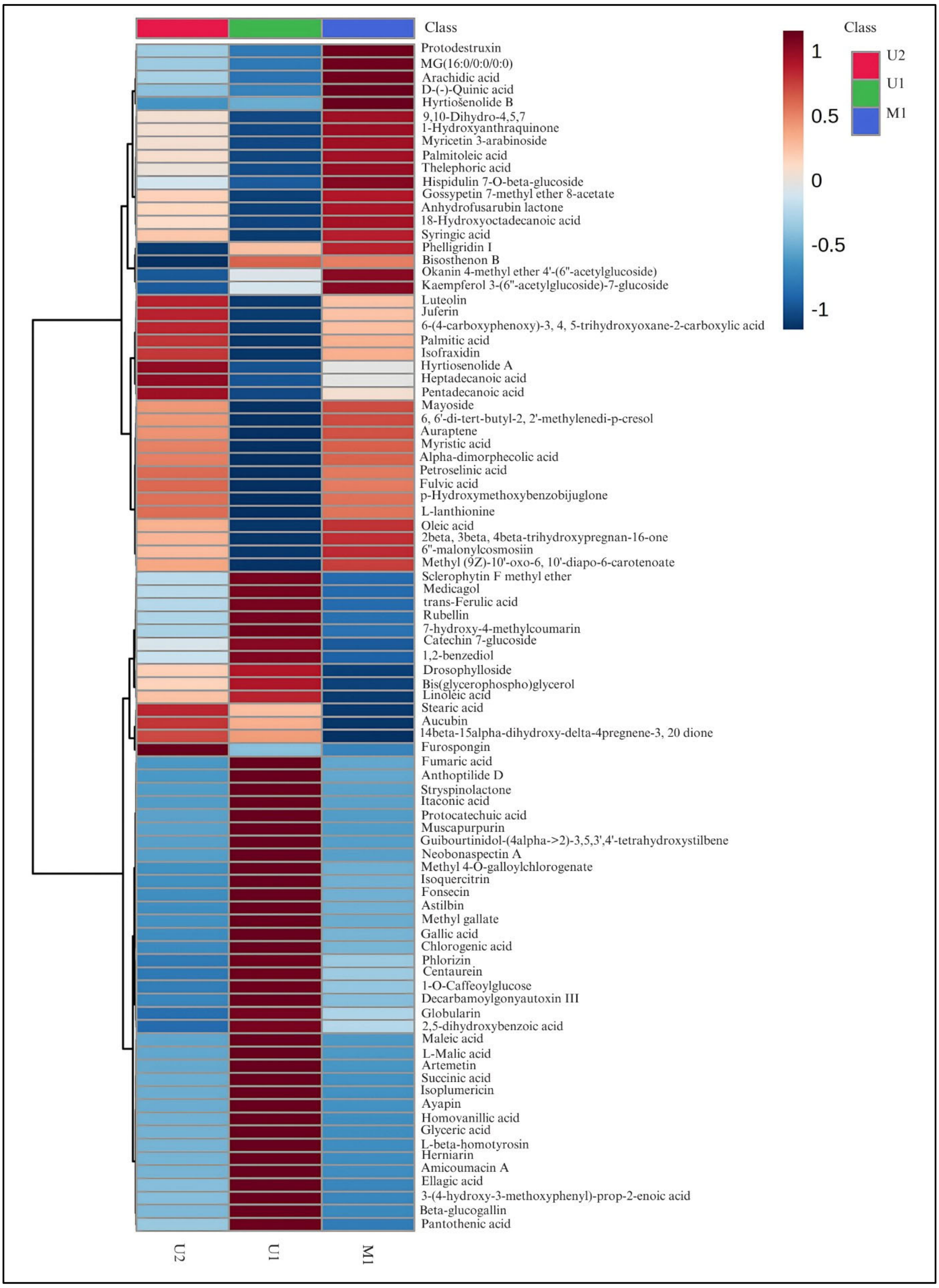

Figure S2. Hierarchical clustering heat map showing the differential distribution of metabolites among the extracts. The color scale represents normalized relative abundance values ranging from –1 (lowest abundance, blue) to +1 (highest abundance, red).

Disclaimer/Publisher's Note: The statements, opinions and data contained in all publications are solely those of the individual author(s) and contributor(s) and not of MDPI and/or the editor(s). MDPI and/or the editor(s) disclaim responsibility for any injury to people or property resulting from any ideas, methods, instructions or products referred to in the content.
